# Analysis of Dynamic Changes of Scallop (*Patinopecten yessoensis*) Adductor Muscle and Mantle by Non-Targeted Metabolomics

**DOI:** 10.3390/foods15030526

**Published:** 2026-02-03

**Authors:** Di Yu, Zuoan Yu, Zhiyu Fu, Lei Qin, Jianan Yan, Long Li, Yujun Liu, Zhiyuan Song, Xiangfeng Liu, Qingzhi Wang, Bailing Chen, Hai Chi, Jie Zheng

**Affiliations:** 1 Dalian Key Laboratory of Genetic Resources Marine Shellfish, Key Laboratory of Protection and Utilization of Aquatic Germplasm Resource, Ministry of Agriculture and Rural Affairs, Liaoning Ocean and Fisheries Science Research Institute, Dalian 116023, China; yudi1986@126.com (D.Y.); abc2033864320@sohu.com (Z.Y.); hkyfzy@126.com (Z.F.); liilong1995@126.com (L.L.); lyj7005@126.com (Y.L.); ultimatun@163.com (X.L.); bailingchen1025@126.com (B.C.); chih@ecsf.ac.cn (H.C.); 2 State Key Laboratory (SKL) of Marine Food Processing & Safety Control, National Engineering Research Center of Seafood, School of Food Science and Technology, Dalian Polytechnic University, Dalian 116034, China; qinlei@dlpu.edu.cn (L.Q.); yjn3vv@163.com (J.Y.); 3 College of Food Science and Engineering, Dalian Ocean University, Dalian 116023, China; songzhiyuan@dlou.edu.cn

**Keywords:** scallop, adductor muscle, mantle, non-targeted metabolomics, harvest period, dynamic metabolites changes

## Abstract

A non-targeted metabolomics technique was employed to investigate the dynamic metabolite changes of scallop (*Patinopecten yessoensis*) adductor muscle (SAM) and scallop mantle (SM) during the early (E), middle (M), and late (L) harvest period from June to July. Many more metabolites were identified from SM (831) than from SAM (231), and 24 and 12 significantly differential metabolites were screened, respectively. Organic acids and derivatives, lipids and lipid-like molecules, and organoheterocyclic compounds were the primary metabolite classes in both SAM and SM. In SAM, the levels of most altered metabolites, such as butyryl carnitine, (±)8-HEPE, 14(S)-HDHA, l-Kynurenine, and l-(−)-Methionine), decreased with the extended harvest time, whereas pipecolic acid and oleamide increased. Conversely, in SM, linoleamide, oleamide, dimethyl citrate, kynurenine, and pipecolic acid declined, while 5,6-Dihydrothymidine, dimethylsulfoniopropionate, and 13(S)-HOTrE increased with a longer harvest time. Pipecolic acid, exhibiting an obvious up-regulated response during the whole harvest period, was found to be the sole differential metabolite shared by SAM and SM. Annotation analysis showed that five metabolites were respectively identified in SAM and SM, and these metabolites were separately related to five metabolic pathways with slight differences among the two tissues. Amino acid metabolism/degradation and fatty acid metabolism were the primary pathways. These findings could provide new insights into the dynamic quality changes of scallops during the harvest period and may play a potential guiding role in the aquaculture and harvest of scallops.

## 1. Introduction

According to the China Fishery Statistical Yearbook [1], shellfish production in 2023 was about 16.46 million tons, accounting for approximately 68.70% of total mariculture production. Scallop (*Patinopecten yessoensis*, *P. yessoensis*), a member of the bivalve mollusk, is native to northern Japan, the Russian Far East, and northern Korea [2]. After being introduced to China in the 1980s, the scallop has been widely cultured in Liaoning and Shandong provinces, and the culture scale has continued to expand [3]. Changhai County, an island in the North Yellow Sea, is the most important culture area for scallops in China [4]. The primary culture mode in this area is floating raft culture and bottom sowing culture [5]. The growth cycle of most raft-cultured scallops is from May in the first year to July in the following year and the primary harvest period spans from June to July in the subsequent year during which the seawater temperature rises gradually.

Scallops, as an important fishery resource, have been attracting much attention from consumers and researchers due to their high nutritional value and unique flavor, such as umami and a sweet taste, as well as their various bioactive substances, etc. Scallops are usually processed into various products because fresh ones are prone to spoilage under the influences of high water content, microorganisms, endogenous enzymes, and external stimuli. Raw materials usually play an important role in the production process of final products. Quality traits and processing characteristics of raw materials have been proven to be closely related to the area of origin [6,7], seasons [8,9,10], species [11], growth stages [12], environmental conditions [13,14], production methods [15], and so on. However, up to now, research about *P. yessoensis* have been mainly focused on aquaculture purification, fresh-keeping, processing, storage, and so on. The nutrition and flavor changes of *P. yessoensis* have been explored in several studies, but the information remains relatively limited. Zhang et al. [16] revealed the seasonal variation of myofibrillar protein thermal stability of *P. yessoensis*, and Liu et al. [17] investigated the nutritional value and flavor differences of *P. yessoensis* (different genders and tissues) based on free amino acids, 5′-nucleotides, and lipids detected by targeted and non-targeted metabolomics.

Metabolomics is a simultaneous qualitative and quantitative analysis method of all small molecular weight metabolites in organisms or cells [18] and is the primary technological means to obtain comprehensive information about metabolites inside and outside cells [19]. Metabolomics has been widely used in the food industry [20]. The characteristics and dynamic changes of food flavor substances and nutrition quality were revealed by metabolomics [21]. In the last few years, the application of metabolomics in marine organisms has increased our understanding of the interactions between certain marine organisms and their survival environment [22]. These studies mainly focused on the seasonal changes of metabolites [23], characteristic metabolites in different habitats and culture methods [24,25], and adaptation analysis of marine organisms to external stress [26,27,28].

In the present study, a time series tracking strategy was adopted, dividing the entire harvesting period into three different stages, the early, middle, and late periods, in order to more accurately understand the dynamic changes of scallops during the harvesting stage. By designing a multi-timepoint dynamic sampling scheme, the dynamic fluctuation characteristics of metabolites and the differential metabolites of scallop adductor muscle (SAM) and scallop mantle (SM) during the harvest period were systematically captured using a non-targeted metabolomics approach. Meanwhile, the metabolic pathways of the selected differential metabolites were further analyzed. The findings can provide a theoretical basis for modeling metabolite–environment relationships, and they will inform future studies on the scallop quality formation mechanism and directly guide marine aquaculture and processing practices.

## 2. Materials and Methods

### 2.1. Materials Collection and Pretreatment

**Figure 1:**
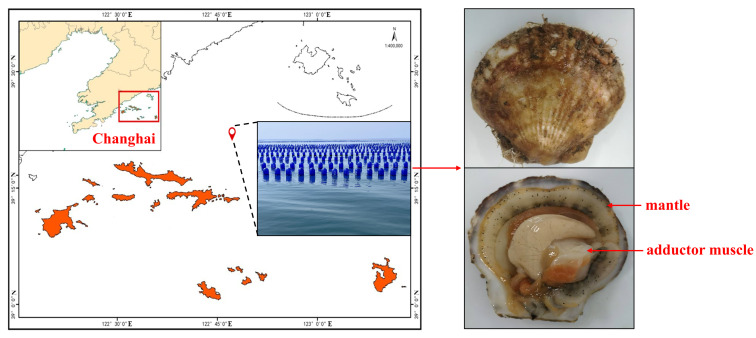
Geographical origin, culture mode, and tissues of scallop (*Patinopecten yessoensis*).

Scallops were collected from the cultural area near Yingjie village, Xiaochangshan Island, Changhai County, Dalian, China (Figure 1) at different harvest times. The species was identified by Cytochrome c Oxidase subunit I (COI) gene sequencing method. After being harvested from the marine cultivation cages, the samples were quickly washed by seawater and then immediately transported to the nearby laboratory within an hour. The biological characteristics, including body weight and shell height, were firstly measured (*n* = 30). The adductor muscle and mantle were subsequently separated from the scallop shell with a scalpel. All the samples were sub-packaged with 10 in each Ziplock bag, transferred to the laboratory located in Liaoning Ocean and Fisheries Science Research Institute, and stored at −80 °C for later analysis. For each sampling time, 120 adductor muscle and mantle of scallops were randomly selected. Every 40 were mixed as a parallel sample, freeze-dried and passed through an 80-mesh sieve for further metabolomics analysis.

### 2.2. Instruments and Chemicals

Ultra-High Performance Liquid Chromatography (Thermo Vanquish) and Q-Exactive HF High Resolution Mass Spectrum are all from Thermo Fisher Scientific (Waltham, MA, USA). Methanol, acetonitrile, and 2-propanol of LC-MS grade were purchased from Merck KGaA (Darmstadt, Germany). Formic acid (purity 99.97 %) was obtained from Xiya Reagent (Shandong, China).

### 2.3. Sample Preparation

Samples were prepared according to the method of Li et al. [29] with slight modifications. Evenly mixed scallop adductor muscle and mantle powder (100 mg of each) were accurately weighed and placed into a 2 mL centrifuge tube, followed by the addition of 1 mL methanol (70%) and steel balls (3 mm). The mixture was firstly ground for 3 min at 70 Hz with a JXFSTPRP-48 fully automatic sample rapid grinder (JingXin, Shanghai, China) and then cooled. Subsequently, the ultrasonic extraction process was performed for 10 min under low temperature and 40 kHz. After centrifugation at 16,000× *g* for 10 min at 4 °C, the supernatant was properly diluted and passed through a 0.22 μm Poly Tetra Fluoro Ethylene (PTFE) filter for instrumental analysis.

### 2.4. LC-MS Analysis

An Ultra-High Performance Liquid Chromatography (UHPLC, Thermo Vanquish, Thermo Fisher Scientific, Waltham, USA) in tandem with a Q-Exactive HF High-resolution mass spectrum (Thermo Fisher Scientific, Waltham, USA) was adopted for metabolites analysis in positive and negative ion mode, respectively. The analytes were eluted and separated by a Zorbax Eclipse C18 chromatographic column (2.1 mm × 100 mm, 1.8 μm) at a constant flow rate of 0.3 mL/min. The mobile phase consisted of 0.1% formic acid (A) and pure acetonitrile (B). The column temperature, injection volume, and autosampler temperature were 30 °C, 2 μL, and 4 °C, respectively. The electrospray ionization (ESI) conditions were set as follows: heater temperature 325 °C, sheath gas flow 45 arb (arbitrary units), aux gas flow 15 arb, sweep gas flow 1 arb, electrospray voltage 3.5 KV, capillary temperature 330 °C, and S-Lens RF Level 55%. The scanning mode was set as follows: full scan (m/z 100~1500) and data-dependent mass spectrometry (dd-MS2, Top- 5); resolution: 120,000 (MS1) and 60,000 (MS2); and collision mode: High Energy Collision Dissociation (HCD).

### 2.5. Statistical Analysis

Compound Discoverer 3.3 was used for retention time correction, peak identification, peak extraction, peak integration, peak alignment, etc. Meanwhile, the Thermo mzCloud online database, Thermo mzValut local database, and ChemSpider database were used for substance identification.

Principal Component Analysis (PCA) and Orthogonal Projections to Latent Structures-Discriminant Analysis (OPLS-DA) were performed with SIMCA 14.1 (Hotelling’s T^2^ test) to investigate the overall metabolite differences among different samples and assess the variability within the same group. The non-orthogonal and orthogonal variables were analyzed to obtain more reliable information about the inter-group differences of metabolites and the correlation degree of experimental groups. OPLS-DA is a supervised discriminant analysis method, which establishes the association model between metabolite expression and sample group by combining the sample variable matrix and classification matrix with the orthogonal partial least squares method. The reliability of the model was checked 200 times, with R^2^X and R^2^Y representing the explanatory rate of the built model for the X and Y matrices, respectively, and Q^2^ representing the predictive power of the model. The differential metabolites were screened based on |log2(Fold Change)| ≥ 1, variable importance in the projection (VIP) value > 1, and *p*-value from the student’s *t*-test < 0.05. PCA and OPLS-DA analyses were performed with SIMCA 14.1. Volcano maps and bubble maps were drawn with GraphPad 9.5.1, and heat maps were drawn on the ChiPlot website. The Kyoto Encyclopedia of Genes and Genomes (KEGG) database was used to annotate the differential metabolites, and the KEGG pathway was enriched by MetaboAnalystR 5.0.

## 3. Results and Discussion

### 3.1. Basic Sampling Information

Detailed sample information is listed in Table 1. As shown in Table 1, the SAM and SM of different harvest times account for about 15% and 9% (on a wet basis) of the total body weight, respectively, and there was no significant difference in this proportion among different harvest times.

### 3.2. Metabolic Profiles of SAM and SM from Different Harvest Time

**Figure 2:**
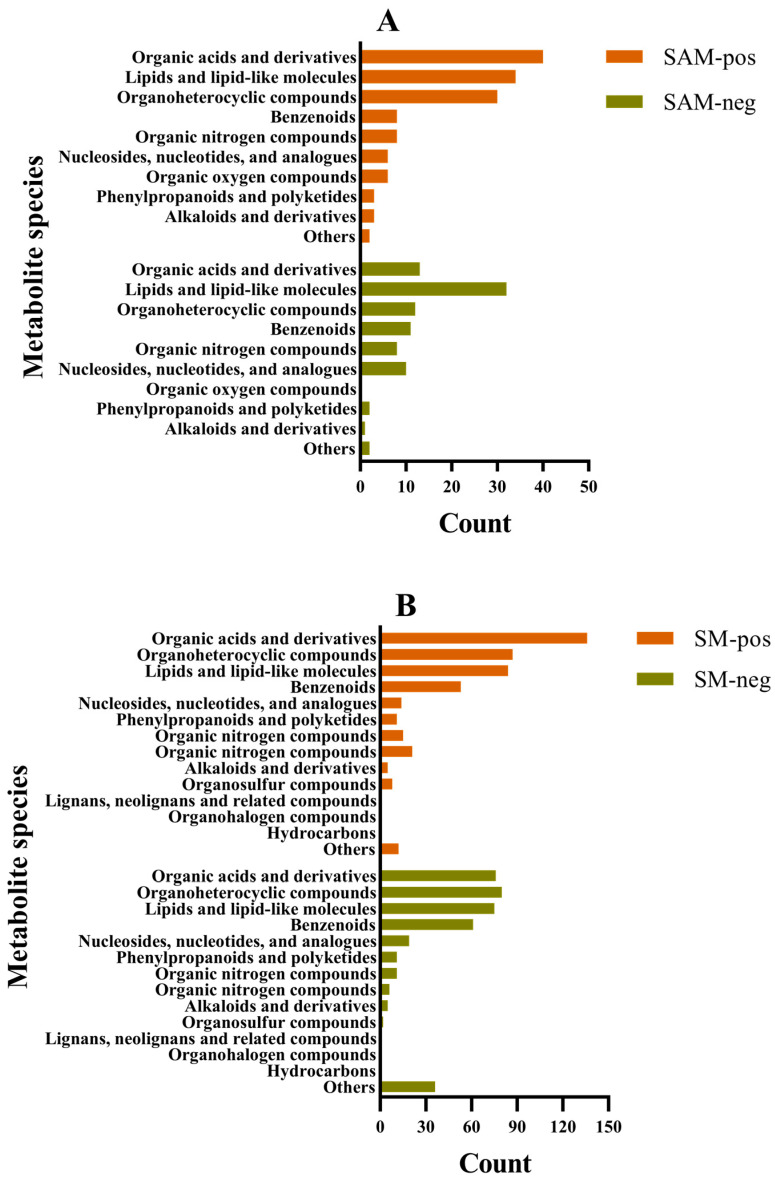
Metabolite species of scallop adductor muscle (**A**) and scallop mantle (**B**) identified in positive and negative ion modes, respectively. SAM: scallop adductor muscle; SM: scallop mantle; pos: in positive ion mode; neg: in negative ion mode; the same below.

A total of 231 metabolites were identified from SAM with 140 in the positive ion mode and 91 in the negative ion mode, and 831 metabolites were simultaneously identified from SM including 446 in the positive ion mode and 385 in the negative ion mode. The identified metabolites of SAM and SM in different ion modes could be divided into different types (10, 9, 11, and 14, respectively) at the super-class level according to the method described by Djoumbou Feunang et al. [30] (Figure 2). Organic acids and derivatives, lipids and lipid-like molecules, and organoheterocyclic compounds were the main metabolite types existing in SAM and SM. In addition, a lot of compounds belonging to benzenoids were also identified.

**Figure 3:**
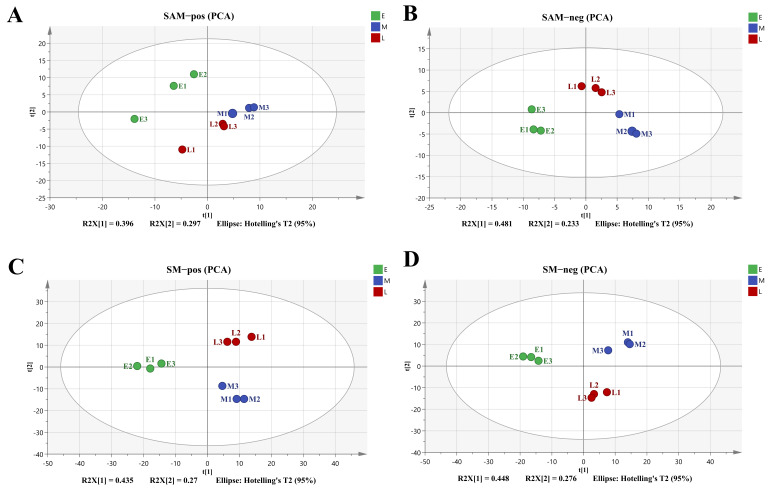
PCA score scatter plots of the differences between scallop adductor muscle and mantle harvested from different periods. E: early harvest period; M: middle harvest period; L: late harvest period. (**A**) adductor muscle in positive ion mode; (**B**) adductor muscle in negative mode; (**C**) mantle in positive ion mode; (**D**) mantle in negative mode.

The PCA method was firstly used to analyze the metabolite differences between SAM and SM harvested at different times, and the score plots for all groups in different ion modes were clearly shown in Figure 3. Obviously, coordinate points of all samples were located in the confidence interval of 95%, and all parallel samples were distributed closely to each other except for SAM in the positive ion mode, indicating that there were remarkable metabolite differences among samples from different harvest times.

**Figure 4:**
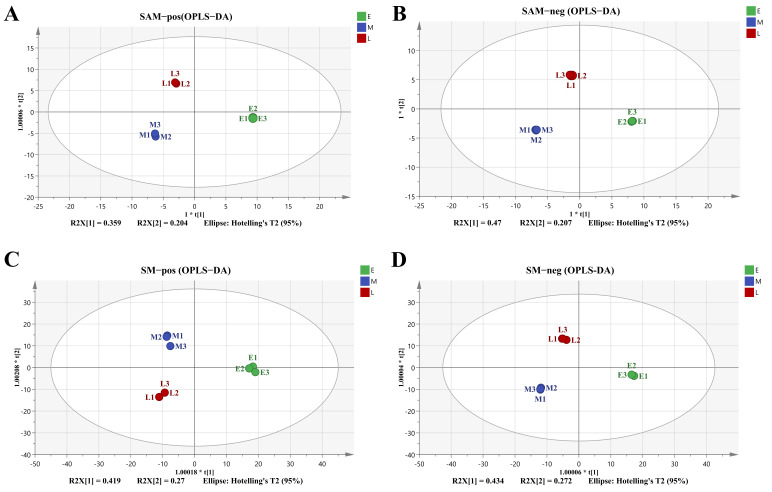
OPLS-DA score scatter plots of the differences between scallop adductor muscle and mantle harvested from different periods. E: early harvest period; M: middle harvest period; L: late harvest period. (**A**) adductor muscle in positive ion mode; (**B**) adductor muscle in negative mode; (**C**) mantle in positive ion mode; (**D**) mantle in negative ion mode.

Due to the limitations of the PCA model [31], a supervised OPLS-DA method was employed to reduce the system noise and extract variable information in order to further amplify the differences between sample groups. The OPLS-DA score plots for all groups exhibited stronger clustering according to the harvest time (Figure 4). Each group was nearly overlapped, and could be distinguished from the other groups. The values of R^2^X (cum), R^2^Y (cum), and Q^2^ (cum) of the built OPLS-DA models (SAM in positive ion mode: R^2^X(cum) = 0.949, R^2^Y(cum) = 0.999, Q^2^(cum) = 0.986; in negative ion mode: R^2^X(cum) = 0.974, R^2^Y(cum) = 1, Q^2^(cum) = 0.986; SM in positive ion mode: R^2^X(cum) = 0.799, R^2^Y(cum) = 0.988, Q^2^(cum) = 0.944; in negative ion mode: R^2^X(cum) = 0.879, R^2^Y(cum) = 0.999, Q^2^(cum) = 0.973, respectively) were valuable for the identification of metabolite differences among different groups.

**Figure 5:**
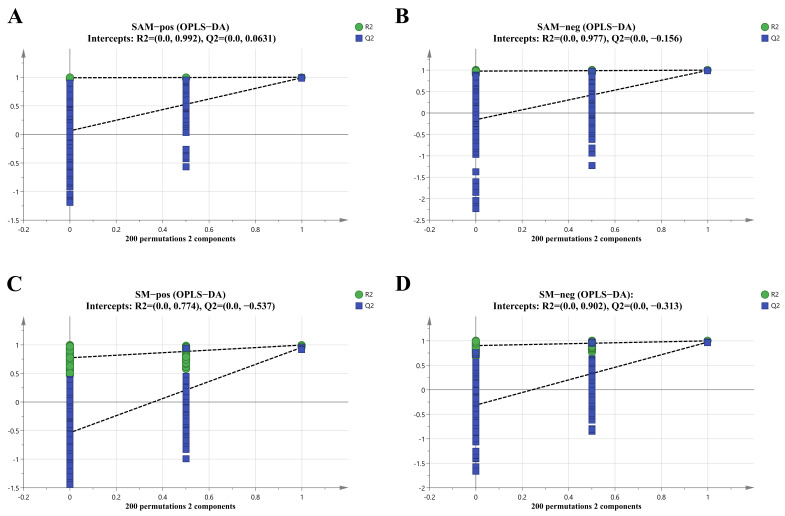
OPLS-DA model permutation test. (**A**) adductor muscle in positive ion mode; (**B**) adductor muscle in negative mode; (**C**) mantle in positive ion mode; (**D**) mantle in negative ion mode.

Moreover, a permutation test was performed (Figure 5) to check the predictability and reliability of the model. No obvious over-fitting was observed according to the evaluation parameters (R^2^ and Q^2^) of the relative models (SAM in positive ion mode: R^2^ = 0.993, Q^2^ = 0.113; in negative ion mode: R^2^ = 0.977, Q^2^ = −0.156; SM in positive ion mode: R^2^ = 0.774, Q^2^ = −0.537; in negative ion mode: R^2^ = 0.900, Q^2^ = −0.351), providing good model validation and accuracy.

### 3.3. Differential Metabolites Analysis of SAM and SM from Different Harvest Times

**Figure 6:**
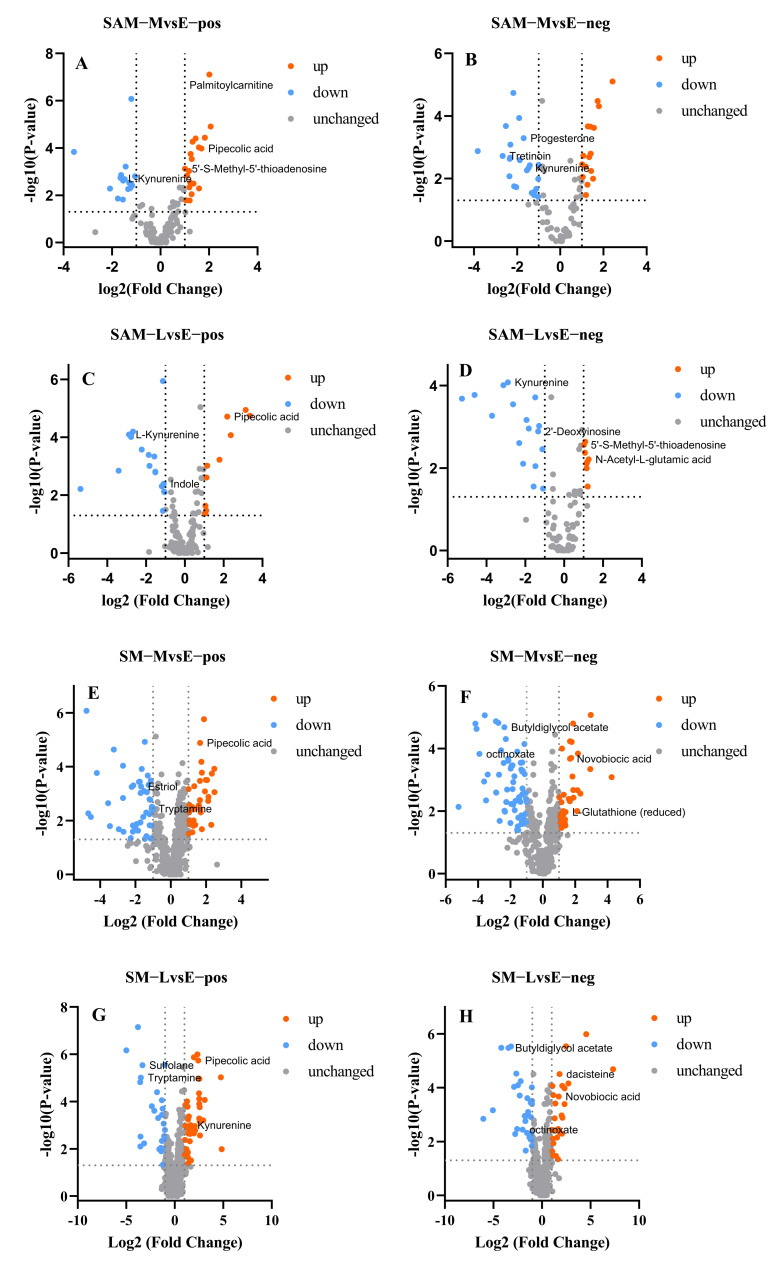
Volcano plots of differential metabolites in scallop adductor muscle and mantle harvested from different periods. (**A**) M vs. E of adductor muscle in positive ion mode; (**B**) M vs. E of adductor muscle in negative ion mode; (**C**) L vs. E of adductor muscle in positive ion mode; (**D**) L vs. E of adductor muscle in negative ion mode; (**E**) M vs. E of mantle in positive ion mode; (**F**) M vs. E of mantle in negative ion mode; (**G**) L vs. E of mantle in positive ion mode; (**H**) L vs. E of mantle in negative ion mode.

The dynamic metabolites changes of SAM and SM from different harvest times (E, M, and L) were analyzed and revealed by multivariate statistical analysis. Metabolite differences were determined by a pairwise comparative analysis method in positive and negative ion modes, and the corresponding volcano plots are shown in Figure 6. Obvious differences could be observed from different sample groups.

Figure 6A–D mainly shows the metabolite differences of SAM. Previous studies have confirmed that the adductor muscle is the motor organ of scallops, responsible for the rapid adduction and abduction of the shell [32]. A total of 74 differential metabolites were identified in MvsE mode, among which 19 were up-regulated and 16 were down-regulated in the positive ion mode (Figure 6A), and 16 were up-regulated and 23 were down-regulated in the negative ion mode (Figure 6B). In LvsE mode, 52 differential metabolites were identified, among which 28 were identified in the positive ion mode including 11 up-regulated and 17 down-regulated (Figure 6C), and 24 were identified in the negative ion mode including 8 up-regulated and 16 down-regulated (Figure 6D). Compared with the adductor muscles, the mantle has more functions such as shell synthesis, respiratory function, movement assistance, and sensation [33,34]. More differential metabolites were screened in SM (Figure 6E–H). Compared to group E, a total of 166 differential metabolites were identified in group M with 77 in the positive ion mode (Figure 6E, 35 up-regulated and 42 down-regulated) and 89 in the negative ion mode (Figure 6F, 30 up-regulated and 59 down-regulated). Meanwhile, 113 differential metabolites were identified in group L with 60 in the positive ion mode (Figure 6G, 37 up-regulated and 23 down-regulated) and 53 in the negative ion mode (Figure 6H, 24 up-regulated and 29 down-regulated). The endogenous metabolites of SAM and SM varied greatly as the growth stage changed. Many studies suggested that high-temperature stress caused by the increase in seawater temperature has a significant impact on the energy metabolism of scallop [35]. There was a certain relationship between flavor and metabolites [36]. Therefore, the dynamic changes of these metabolites may play an important role in the formation of the quality characteristics of scallops during the harvest period.

Indicators of |log2(Fold change)| ≥ 1, VIP value > 1, and *p*-value < 0.05 were used to further screen the important differential metabolites. For SAM, 12 significantly differential metabolites were identified with seven in MvsE mode and five in LvsE mode, respectively, in comparison with group E (Table 2). Most of differential metabolites were identified in positive ion mode (10 of 12) and showed down-regulated responses (7 of 12). As observed from Table 2, pipecolic acid exhibited up-regulated response in the two comparative modes, while butyryl carnitine (an isomer of 920) and L-Kynurenine exhibited down-regulated responses. Additionally, 24 differential metabolites were screened from SM in comparison with group E (Table 3), among which almost half metabolites were identified in the positive ion mode and showed up-regulated responses. The metabolites screened in MvsE mode were more than in LvsE mode (15 vs. 9). L-glutathione (reduced) and 5,6-dihydrothymidine were both detected in positive and negative ion modes in the same comparative mode. 13(S)-HOTrE was shared by the two comparative modes and showed down-regulated responses during the harvest period, while another mutually differential metabolite, pipecolic acid, exhibited an up-regulated response. In summary, the unique differential metabolite, pipecolic acid, was found in the two comparative modes either in SAM or in SM. Pipecolic acid, a metabolite of lysine that existed in various organisms, was considered to be an important biomedical marker for peroxisomal-related disorders including Zellweger’s syndrome, etc., and played an important depressive role in the central nervous system [37]. It has also been proven that pipecolic acid has regulatory effects on proteolysis and protein synthesis in C2C12 myotubes [38] and inhibitory effects on angiotensin I-converting enzyme [39]. In addition, pipecolic acid, as one of the natural precursors, may lead to the formation of mepiquat under typical baking/roasting temperatures commonly occurring in the food processing industry [40]. Up to now, information about the structure type, existence form, origin source, and potential role of pipecolic acid in scallops was rarely known and needed to be further studied in the future.

**Figure 7:**
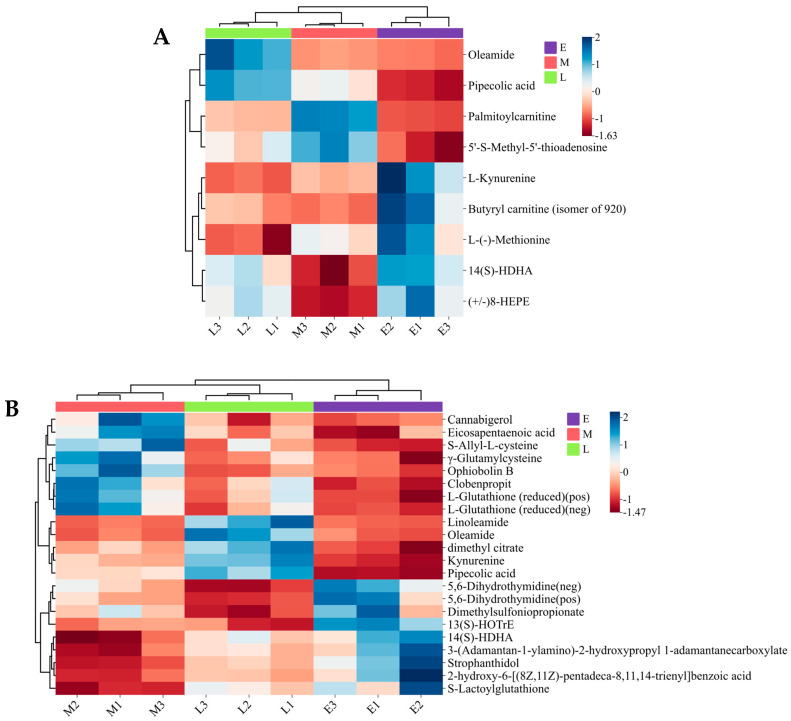
Clustering heatmap of metabolites in scallop adductor muscle (**A**) and mantle (**B**) harvested from different periods.

To simply and intuitively observe the relative content and clustering relationship of important differentially expressed metabolites in the experimental groups, cluster analysis was performed, and labeled metabolites were all presented in the form of heat maps for SAM (Figure 7A) and SM (Figure 7B). In SAM, levels of most altered metabolites, including butyryl carnitine (isomer of 920), (+/−)8-HEPE, 14(S)-HDHA, L-Kynurenine, and L-(−)-Methionine, were decreased with the extension of the harvest period, while the levels of pipecolic acid and oleamide increased. Metabolites of SM were relatively more diversified than those of SAM, and correspondingly, many more metabolites were observed to be changed in SM than in SAM. Moreover, linoleamide, oleamide, dimethyl citrate, kynurenine, and pipecolic acid were found to be decreased while 5,6-Dihydrothymidine dimethylsulfoniopropionate, and 13(S)-HOTrE increased with the extension of harvest time in SM.

Interestingly, the contents of pipecolic acid and oleamide changed in opposite trends in SAM and SM with the extension of the harvest time, and this phenomenon may be closely related to the metabolic adaptation mechanism of scallops in response to environmental challenges [41,42]. The metabolic response of scallops to heat stress and hypoxia stress has been proven to be tissue-specific [43], which depends on the role of different tissues in energy distribution [44]. The adductor muscle and mantle are two distinct tissues of scallop, and perform obviously different physiological functions during the breeding process of scallop. Previous study demonstrated that warming had an effect on carbohydrate metabolism, amino acid metabolism, and other amino acid metabolic pathways in gills [45]. The metabolic rate of SAM increases with the rise of temperature, manifested as an increase in oxygen consumption and ammonia excretion rate [46]. However, the metabolic responses of mantle to such changes are still poorly understood.

### 3.4. Metabolic Pathways Analysis of Differential Metabolites Identified from SAM and SM

**Figure 8:**
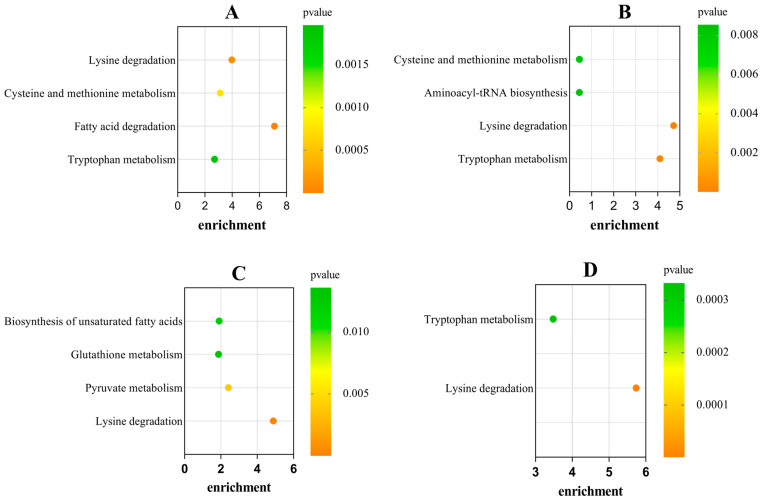
KEGG enrichment analysis of differential metabolites in scallop adductor muscle and mantle. (**A**) SAM−MvsE; (**B**) SAM−LvsE; (**C**) SM−MvsE; (**D**) SM−LvsE.

The KEGG database was used for an annotation analysis of differential metabolites, and an enrichment analysis was also performed to identify the metabolic pathways with significant changes in expression levels between different groups (Figure 8). In the MvsE mode of SAM, four different metabolites, containing pipecolic acid, 5′-S-Methyl-5′-thioadenosine, palmitoylcarnitine, and L-Kynurenine, were successfully matched in the KEGG database (Figure 8A), and meanwhile, pipecolic acid, S-Lactoylglutathione, γ-Glutamylcysteine, and eicosapentaenoic acid were also matched in the mantle (Figure 8C). The enrichment pathways of differential metabolites in SAM were mainly related to “lysine degradation”, “cysteine and methionine metabolism”, “fatty acid degradation” and “tryptophan metabolism”, while the pathways in SM were related to “biosynthesis of unsaturated fatty acids”, “glutathione metabolism”, “pyruvate metabolism” and “lysine degradation”. In the LvsE mode, L-(−)-methionine, pipecolic acid, and L-kynurenine were successfully matched in SAM (Figure 8B), and the pathways were related to “cysteine and methionine metabolism”, “aminoacyl-tRNA biosynthesis”, “lysine degradation” and “tryptophan metabolism”. In the LvsE mode of SM, pipecolic acid and kynurenine were matched in the KEGG database (Figure 8D) and were principally involved in “tryptophan metabolism” and “lysine degradation” pathways. In general, lysine degradation was the sole mutual enrichment pathway for both SAM and SM, and this pathway usually occurs through α-transamination or α-deamination of lysine [47].

**Figure 9:**
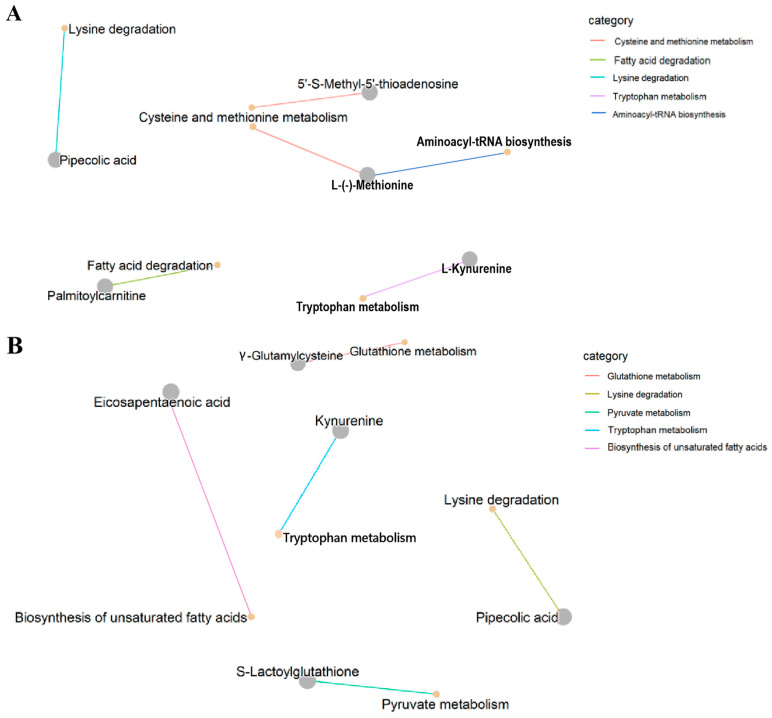
Connect plots of different metabolites in scallop adductor muscle (**A**) and mantle (**B**). The yellow node represents the name of the pathway; the connected gray node represents the specific metabolite annotated to the pathway; the color of the connecting line represents different pathways.

Metabolic pathways from the KEGG database and the corresponding metabolites were summarized and shown in a network graph (Figure 9), which enabled the rapid discovery of metabolic pathways and compounds associated with specific biological processes, and could also help determine the metabolites involved in the regulation of these pathways. Metabolic regulation is a comprehensive process involving a variety of metabolites, proteins, and genes [48]. The KEGG pathway results showed that the dynamic changes of five metabolites (pipecolic acid, 5′-S-Methyl-5′-thioadenosine, palmitoylcarnitine, L-(−)-Methionine, and L-Kynurenine) in SAM during the harvest period were matched to different pathways including amino acid metabolism/degradation, fatty acid degradation and aminoacyl-tRNA biosynthesis. Differential metabolites are all related to amino acid metabolism/degradation (cysteine, methionine, lysine, and tryptophan) except palmitoylcarnitine, which is associated with fatty acid degradation. In addition to the amino acid metabolism/degradation (pipecolic acid and kynurenine) and unsaturated fatty acid biosynthesis (Eicosapentaenoic acid), the pathways of changed metabolites in SM were also related to pyruvate metabolism (S-Lactoylglutathione) and glutathione metabolism (γ-Glutamylcysteine). S-Lactoylglutathione is an intermediate in the conversion of methylglyoxal to pyruvate [49]. Glutathione is a tripeptide composed of glutamic acid, cysteine, and glycine [50]. γ-Glutamylcysteine was a dipeptide formed by the connection of glutamic acid and cysteine through a γ-peptide bond, and it serves as the precursor for the synthesis of glutathione [51]. Pipecolic acid, related to lysine degradation, was identified as a differential metabolite in both SAM and SM. Amino acids, related to the above metabolic pathways, have different taste characteristics. Cysteine has an acidic taste, while methionine, lysine, and tryptophan are bitter amino acids [52,53]. Pyruvate is an important intermediate of glucose metabolism in all biological cells, and fatty acids are precursors of flavor substances and play an important role in food taste formation [54]. These results indicated that there may be taste changes in SAM and SM during the harvest period.

Temperature was one of the main environmental stressors of scallops [55]. It has been reported that the optimum growth temperature of scallops was 10 °C to 15 °C [56]. In previous studies, the energy regulation and metabolism, intestinal histology, and microbiota of scallops under high-temperature stress had been proven [8,35,57]. During the sampling period of the present study, the seawater temperature in the culture area was the most variable environmental factor, being observed to increase from 14.87 ± 3.49 °C to 19.55 ± 3.45 °C. It was deduced that the obvious metabolite changes may be mainly due to the change in environmental temperature.

## 4. Conclusions

Great metabolic changes of SAM and SM harvested at different time were observed, and the metabolic responses of SM were more obvious than SAM due to its metabolite diversity. Pipecolic acid was screened to be the primary differential metabolite shared by SAM and SM in all comparative modes and was correspondingly matched to the lysine degradation pathway. Pipecolic acid showed the potential to act as a metabolite biomarker for scallop growth and harvest in the future, but further specific verification is necessary. The present results could further deepen the comprehensive understanding of the metabolic mechanism and quality formation mechanism of scallops during the whole harvest period, and could also provide valuable insights and guiding effects for scallop mariculture and processing.

## Figures and Tables

**Table 1:** Detailed information for scallops captured from different harvest times (on a wet basis).

Sample Code	Sampling Date	Body Weight (g)	Shell Height (cm)	Adductor Muscle Weight (g)	Mantle Weight (g)
E (Early harvest period)	22 June 2020	59.46 ± 7.36 ^a^	77.98 ± 4.03 ^a^	8.72 ± 1.45 ^a^	4.84 ± 0.62 ^a^
M (Middle harvest period)	8 July 2020	47.08 ± 5.91 ^b^	71.47 ± 4.05 ^b^	6.92 ± 1.26 ^b^	4.35 ± 0.76 ^b^
L (Late harvest period)	21 July 2020	41.66 ± 5.91 ^c^	73.18 ± 3.83 ^b^	6.40 ± 1.32 ^b^	3.84 ± 0.64 ^c^

Mean values ± standard deviation (*n* = 30). Means within same column with different letters are significantly different at *p* < 0.05.

**Table 2:** Significantly differential metabolites in SAM captured from different harvest times.

Serial Number	Metabolites	Retention Time	Mode	Log2(Fold Change)	M vs. E			L vs. E		
					VIP	*p*-Value	Changes	VIP	*p*-Value	Changes
1	Pipecolic acid	1.084	POS	1.69	1.44432	0.0001057	up			
2	Butyryl carnitine (isomer of 920)	2.856	POS	−1.48	1.3698	0.0020939	down			
3	5′-S-Methyl-5′-thioadenosine	4.321	POS	1.01	1.3455	0.0007483	up			
4	Palmitoylcarnitine	12.261	POS	2.02	1.40513	0.0000001	up			
5	L-Kynurenine	2.078	POS	−1.61	1.01091	0.0019654	down			
6	(+/−)8-HEPE	12.830	NEG	−1.91	1.43588	0.0001163	down			
7	14(S)-HDHA	13.674	NEG	−1.88	1.24858	0.0026000	down			
8	L-(−)-Methionine	1.092	POS	−1.02				3.62301	0.008537	down
9	Pipecolic acid	1.084	POS	2.19				1.98954	0.000019	up
10	Butyryl carnitine (isomer of 920)	2.856	POS	−1.05				1.31286	0.007920	down
11	L-Kynurenine	2.078	POS	−2.88				1.20831	0.000080	down
12	Oleamide	16.833	POS	3.14				1.24454	0.000011	up

**Table 3:** Significantly differential metabolites in SM harvested from different periods.

Serial Number	Metabolites	Retention Time	Mode	Log2(Fold Change)	M vs. E			L vs. E		
					VIP	*p*-Value	Changes	VIP	*p*-Value	Changes
1	L-Glutathione (reduced)	1.094	POS	1.06	5.9247	0.0097821	up			
2	Pipecolic acid	1.088	POS	1.66	2.23035	0.0000131	up			
3	Clobenpropit	1.096	POS	1.3	1.89725	0.0098753	up			
4	S-Lactoylglutathione	1.262	POS	−1.03	1.35872	0.0039022	down			
5	Ophiobolin B	15.237	POS	1.6	1.67145	0.0026961	up			
6	S-Allyl-L-cysteine	1.451	POS	1.06	1.07241	0.0026232	up			
7	3-(Adamantan-1-ylamino)-2-hydroxypropyl 1-adamantanecarboxylate	13.539	POS	−3.45	1.04409	0.0161008	down			
8	Y-Glutamylcysteine	1.096	POS	1.07	1.05212	0.0136123	up			
9	Eicosapentaenoic acid	15.594	NEG	1.09	5.96467	0.0126221	up			
10	L-Glutathione (reduced)	1.093	NEG	1.4	5.67072	0.0107798	up			
11	14(S)-HDHA	13.677	NEG	−1.96	2.83897	0.0059420	down			
12	2-hydroxy-6-[(8Z,11Z)-pentadeca-8,11,14-trienyl]benzoic acid	13.544	NEG	−3.51	2.32542	0.0045900	down			
13	13(S)-HOTrE	12.498	NEG	−1.33	1.68454	0.0262136	down			
14	Cannabigerol	16.373	NEG	1.08	1.15361	0.0226854	up			
15	Strophanthidol	13.029	NEG	−2.71	1.12071	0.000687484	down			
16	5,6-Dihydrothymidine	0.822	POS	−1.33				2.80947	0.0046332	down
17	Dimethylsulfoniopropionate	0.784	POS	−1.4				2.0798	0.0127090	down
18	Pipecolic acid	1.088	POS	2.43				2.75414	0.0000018	up
19	Oleamide	16.833	POS	2.58				2.61837	0.0006895	up
20	Linoleamide	15.22	POS	3.09				1.91422	0.0000854	up
21	Kynurenine	0.84	POS	1.64						
22	13(S)-HOTrE	12.498	NEG	−2.56				2.10714	0.0000205	down
23	5,6-Dihydrothymidine	0.821	NEG	−1.43				1.04241	0.0013745	down
24	dimethyl citrate	0.826	NEG	1.31				4.80666	0.0233058	up

## Data Availability

The original contributions presented in the study are included in the article, further inquiries can be directed to the corresponding authors.
